# Co‐production and adaptation of a prison‐based problem‐solving workbook to support the mental health of patients housed within a medium‐ and low‐secure forensic service

**DOI:** 10.1111/hex.13997

**Published:** 2024-02-23

**Authors:** Amanda E. Perry, Heather Baker, Anne Aboaja, Lindsey Wilson, Sarah Morris, Patient Public Involved Service User

**Affiliations:** ^1^ Department of Health Sciences University of York York Fulford UK; ^2^ Tees Esk and Wear Valleys NHS Foundation Trust Darlington West Park UK

**Keywords:** co‐production, feasibility, forensic, problem solving, secure in‐patient services

## Abstract

**Introduction:**

Problem‐solving skills (PSS) help to provide a systematic approach to dealing with and managing complex problems. The overall aim of this study was to assess the acceptability and feasibility of developing and adapting a prison‐based PSS  workbook for adults within a medium‐ and low‐secure hospital.

**Method:**

We used the Medical Research Council framework in our participatory mixed methods study incorporating an adapted survey (to identify what types of problems people experience in secure hospitals), a series of three interactive workshops (to co‐produce two case study examples for a workbook) and we gathered feedback from patients and hospital staff on the acceptability and feasibility of the workbook. Data from the survey were used to inform the case study examples, and the feedback from patients and hospital staff was descriptively summarised and the results consolidated.

**Results:**

In total, 82 (51%) patients took part in the survey; 22 patients and 49 hospital staff provided feedback on the workbook. The survey results indicated that patients regularly experience problems while in the hospital. Patients reported problems relating to restrictions of freedom and boredom. The workshops produced two case studies for the workbooks, with mainly positive patient and staff feedback. More work is required to improve the visual representation of the characters in the case studies, the amount and content of the language and the mechanism of the intervention delivery.

**Conclusion:**

The adaptation process proved acceptable and feasible to both patients and staff. The co‐production methodology for the workbook and feedback from patients and staff was an effective way of iteratively refining the materials to ensure that they were both meaningful and acceptable to staff and patients. Subsequent work is required to develop the workbook and evaluate the feasibility of the intervention delivery, recruitment rates, uptake and adherence to the PSS using a randomised controlled trial.

**Patient or Public Contribution:**

At each stage of the project consultation with patients and/or hospital staff was involved.

## 1 INTRODUCTION

There are currently 60 medium secure units providing up to 7500 NHS‐commissioned inpatient beds in England.^1^ Such units provide access to a range of therapeutic environments that assess, treat and facilitate the recovery of adults who present a serious risk of harm to themselves and others.^2^, ^3^ The current average estimated length of continuous stay in a medium secure hospital setting is over 14 years^4^ (greatly exceeding the recommended 2‐year stay), resulting in nearly 10% of all UK public expenditure spent on this provision of healthcare.^5^, ^6^, ^7^

The treatment and delivery of psychosocial and psychological interventions in secure hospitals are well‐documented.^3^, ^8^, ^9^ The outcomes of a meta‐analysis and systematic review of 14 studies conducted in these settings seem to favour treatment over the comparator condition. Intervention improvements included a greater awareness of self and one's own mental health problems,^10^ a reduction in a perceived ability to benefit from crime, and improvements in ward behaviour and problem‐solving capabilities.^9^, ^11^

Psychological and psychosocial interventions, such as cognitive and dialectical behavioural therapy (CBT and DBT), are cited in the current National Institute of Health and Care Excellence Guidelines and is considered to be the ‘gold standard treatment’ for schizophrenia, depression and anxiety, as well as personality disorder.^11^, ^12^ Both CBT and DBT require active participation from the patient often incorporating problem‐solving skills (PSS) to achieve their goal of improved functioning, symptom reduction and maintenance or remission of the disorder.^13^, ^14^ Clinical trials and a meta‐analysis of PSS delivered through problem‐solving interventions (PSI) highlight the potential to reduce symptoms of depression, anxiety, self‐harm behaviour and improve quality of life. Such skills have been used by several professional and lay person groups in a range of community and prison settings.^15^, ^16^, ^17^, ^18^

In prisons, PSIs have shown positive benefits to those in custody (e.g., increased confidence, empowerment and an improved ability to address their own problems), improved prisoner‐staff relationships, and a reduction in repeat self‐harm behaviour.^16^, ^18^ This research provides an opportunity for further adaptation where allied but different settings (e.g., secure hospitals) house populations of people who display similar levels of mental health, self‐harm and suicidal behaviour to their custodial counterparts.^19^

Intervention adaptation plays an important role in encouraging stakeholder buy‐in and adherence to treatment. Like the prison custodial environment, people living within the constraints of the secure locked hospital face similar challenges. For example, acknowledging that the process of problem‐solving is likely to be restricted by resources and accessibility to the support of family and friends^20^ who and how people ask for help when faced with a problem is an important consideration in how the intervention will be utilised. The natural authoritarian environment and power dynamic in the relationship presented between the prison officer and person in custody compared to the nurse–patient relationship is likely, to enable different mechanisms for delivery, implementation and acceptability of the intervention, which requires engagement from the patient and staff team.^9^, ^10^

Studies have found several factors affecting adherence, including that of the ‘place’ in which the intervention is delivered and the type of health diagnosis.^21^, ^22^, ^23^ Coupled with limited healthcare resources and tighter funding constraints, it is important to address the fundamental questions about the feasibility, acceptance and sustainability of treatment implementation to ensure that resources are used in the best possible way.^24^, ^25^, ^26^, ^27^, ^28^ In 2000, the UK Medical Research Council (MRC) produced guidelines for developing and evaluating complex interventions.^28^ These promote the notion that service user and staff involvement are crucial during all stages of intervention development, however, use of these technics in secure hospital settings are scarce.^29^, ^30^, ^31^, ^32^

For these reasons, this study aimed to understand more about the problems experienced by patients in a low‐ to medium‐secure hospital; co‐produce two new case studies and adapt the prison‐based PSS workbook and consult with patients and staff about the feasibility and acceptability of using the workbook in the hospital setting.

## 2 METHODS

### 2.1 Prior research work

This work stems from a larger feasibility study aimed at developing a problem‐solving training package for prison staff (in four male and female adult prisons) to support people in custody at risk of self‐harm and suicidal behaviour.^16^ Our initial work used an existing seven‐step community‐based problem‐solving model,^18^ which we adapted with service users to provide contextualised case studies, workbooks and a training package. Promising pre‐ and posttest results showed reductions in symptoms of depression and incidence of self‐harm. In subsequent work, the seven‐step model was amended to a six‐step model providing a consistent approach between the study intervention and the Her Majesty's Prison and Probation Service ‘Thinking Skills Program’.^33^ We tested a peer‐led delivery model whereby people in custody were used to deliver the skills to their peers.^18^ As part of an audit, we used a survey to learn more about the problems people experienced in custody and who they would share their problems with and their use of the scheme.^34^ It is this survey and workbook that we adapted to patients in this current study.

### 2.2 Setting and participants

The research took place in a secure hospital in the North of England. Seventeen secure (forensic) wards were invited to take part in the study. The wards provide healthcare for up to 175 male and female adults > 18 years of age, with a mental health disorder, learning disability or diagnosis of autism. One patient service user who had previous experience of contributing to other research projects was approached to identify if they were willing to support our piece of work. This patient reviewed the application proposal, joined the group as a member of our research team and is an author of this paper. The patient made valuable contributions at all stages of the project and because of this engagement, we made changes to the initial design of the study.

### 2.3 Project objectives

**Figure 1:**
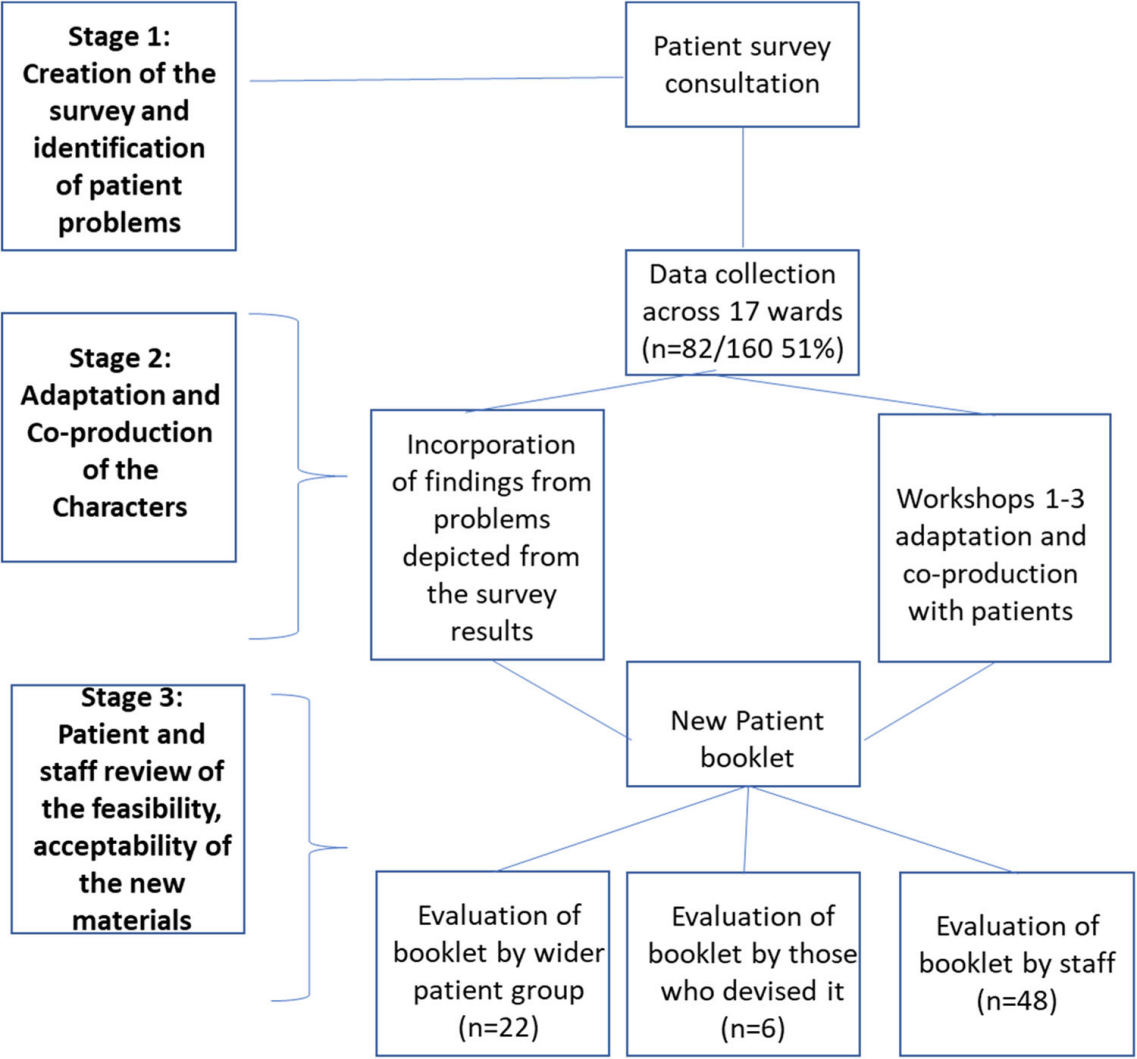
Overview of the study process.

The project (Figure 1) had three key objectives: (i) to identify the problems experienced by patients in the hospital and who they preferred to speak to about them, (ii) to facilitate a small group of patients to co‐produce two case study examples for the new PSS workbook and (iii) to gather feedback through review and consultation with the hospital staff and patients about the feasibility and acceptability of the workbook.

#### 2.3.1 The adaptation of the survey design

The survey design was based on the aforementioned work examining problems faced by people in custody^16^, ^18^ (see Supporting Information S1: Appendix A). During August and October 2021, the adaptation of the prison‐based survey was conducted through a series of virtual patient group meetings with up to six patients. Each consultation group met once a month to review the iterative improvements to the design and wording of the survey. Each iteration was fed back to the group until no more changes were required. A summary of the process and the content of the survey were circulated to the hospital staff and the Hospital Forensic Research Group. This aided the transparency of the work and increased the awareness of the project amongst staff members.

Changes in the wording of questions were used to reflect the medium secure hospital environment (e.g., the wording of ‘wing’ was changed to ‘ward’). In addition, an extra response ('restrictions and freedom’) was added based on the consultation feedback. Two questions (questions 5 and 6) were removed and deemed not applicable. The resulting questionnaire had four questions covering: (i) the experience of problems in the hospital, (ii) the types of problems experienced, (iii) who people approached and talked to about their problems in the hospital and (iv) how often these problems occurred (to understand the extent to which problems were addressed or were an ongoing concern for patients). Responses were multiple choice tick boxes and free text with space to add comments. Data were also collected on gender, age range, ethnicity, ward and length of time in the hospital (see Supporting Information S1: Appendix B).

#### 2.3.2 Dissemination of the patient survey

Between 8 November 2021 and 29 March 2022 and across seven data collection points, data were collected from a voluntary, self‐selecting sample of patients who were on the ward at the time of data collection. Typically, the questionnaire was read to the patient on a one‐to‐one basis and the responses and feedback were recorded verbatim by A. E. P. and H. B.

#### 2.3.3 Co‐production of the case studies for the PSI workbook

Two, 2‐h patient workshops (held on 18 November and 9 December 2021) were used to produce the two case studies for the existing prison workbook (see Supporting Information S1: Appendix C). The workshops introduced the concept of problem‐solving and had patients develop a ‘my life story’ poster. The process of developing the posters involved patients using a series of cut‐out magazine pictures, which represented different aspects of societal life (e.g., houses, activities, pets, food, gardens, cars and hobbies). Patients identified with relatable pictures to create a representation of their own lives. Once complete, patients were asked to supplement the poster by adding a narrative text and/or singular words to depict their own life experiences.

**Figure 2:**
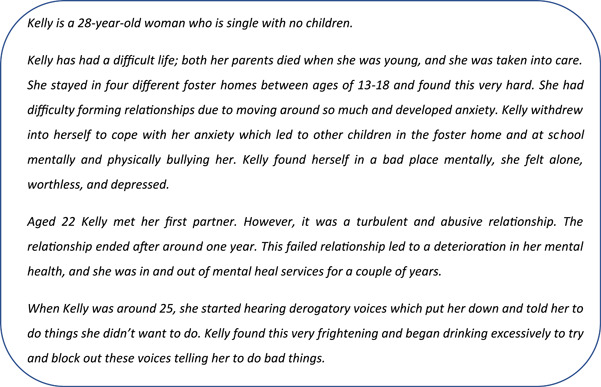
Example of female character story.

**Figure 3:**
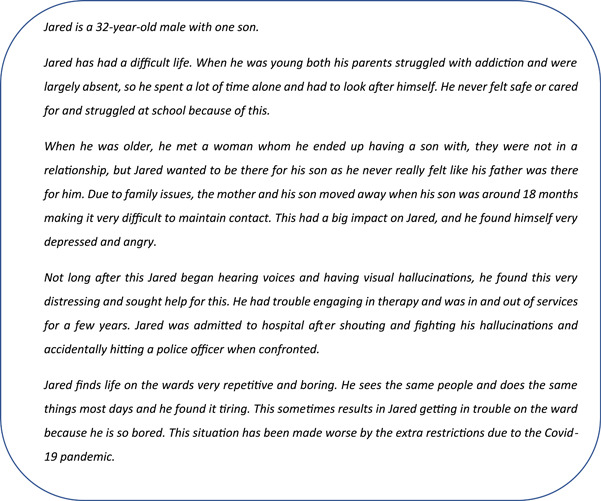
Example of male character story.

Using the words and narrative text on the posters, a second semistructured template was devised by the chief investigator (A. E. P.). The template was used to encourage a full account of an individual's description and to ensure that the same type of information was provided for each story. The semistructured template included information on their experiences in childhood (e.g., Who did you grow up with? Did you feel safe?), adulthood (e.g., tell me about your family, job, health) and what led them to the hospital (e.g., triggers, feelings, an incident). The semistructured information gathered in the template was then combined with poster information and the data collected from the survey. The most prevalent problems reported in the survey (i.e., patient boredom and loneliness) were chosen as the ‘problem topics’ for the case study examples. This resulted in one ‘female’ (referred to as Kelly) and one ‘male’ (referred to as Jared) case study for the workbook. By using this method, patient anonymity was upheld throughout (see Figures 2 and 3).

#### 2.3.4 The consultation review of the adapted workbook with patient and hospital staff

At the third and final workshop patients who had helped devise the workbooks were presented with the new adapted case studies. A feedback form consisting of five questions, and a Likert scale from 1 (strongly disagree) to 5 (strongly agree) was provided to patients at the end of the session. Patients were asked to rate the workbook using the feedback form (see Supporting Information S1: Appendix D). Because this patient group was small with a natural vested interest in the use of the workbook, we gathered feedback, during September and October 2022 from a wider group of patients who had not had any prior involvement and a group of hospital staff.

Patients in all except for two wards (which were deemed not appropriate to approach due to the severity of the patient's illness) were approached by members of the research team to review the workbook and complete the feedback sheet. A voluntary, self‐selecting group of patients provided feedback with a member of the research team H. B. and/or L. W. present. Hospital staff also provided their feedback through staff meetings, personal contacts and email messages. Staff were asked to review the workbook and return the feedback form to a member of the research team L. W.

#### 2.3.5 Data analysis

The survey results were summarised descriptively, and each set of problems was recorded by gender. The data from the review and consultations were evaluated using the mean score between the staff and patient groups at 95% significance testing at *p* < 0.05. The Fisher's exact test was used where the sample size was too small for use with alternative parametric testing. Qualitative comments (gathered from the free‐text qualitative responses in the feedback) were summarised to represent staff and patient views; no formal thematic framework for the analysis was used.

## 3 RESULTS

### 3.1 Patients' experiences and problems reported in a secure hospital setting

**Figure 4:**
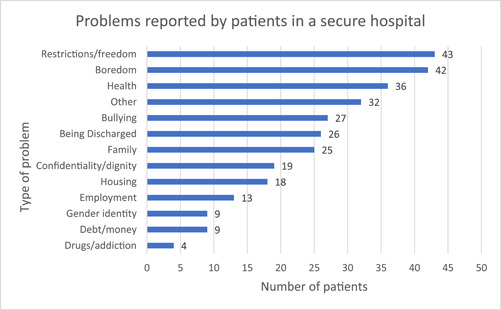
Problems reported by patients in a secure forensic hospital.

In total, 82 out of 160 patients (51%) responded to the survey. Overall, the most reported problems (Figure 4) were those relating to restrictions and freedom of movement (*n* = 43 [52%]); boredom (having a lack of activity) (*n* = 42 [51%]) and problems relating to health (*n* = 36 [43%]). Patients reported a preference to talk to staff about their problems (*n* = 63, [76%]); followed by family members (*n* = 50, [60%]); other patients (*n* = 30, [36%]) and chaplaincy staff (*n* = 15, [18%]). Just under half of all problems were reported as ongoing, (*n* = 39, [47%]); within the last 3–6 months (*n* = 11, [13%]) or rarely happening (*n* = 28, [34%]).

Females (*n* = 21/83, [25%]) were more likely than males (*n* = 61/83, [75%]) to rate health concerns as a problem (*n* = 12, [57%] vs. *n* = 24, [39%]). Males were more likely than females to report restrictions about movement and freedom within the hospital site (*n* = 10, [48%]; vs. *n* = 33, [54%]) and being bored (*n* = 35, [57%]). Females were significantly more likely to speak to a member of the chaplaincy team in comparison with males (males *n* = 8 [13%] vs. females *n* = 7 [33%], *p* = 0.038).

Both males and females preferred talking to staff (males *n* = 16, [76%] vs. females *n* = 47, [77]%); followed by family members (males *n* = 13, [62%] vs. females *n* = 37, [61%]); other patients (males *n* = 5, [24%]vs. females *n* = 25, [41%]) and friends (males *n* = 4, [19%] vs. females *n* = 20, [33%]). Females (males *n* = 12, [57%] vs. females *n* = 27, [44%]) were more likely than males to report problems as ‘ongoing’ (Table 1).

**Table 1:** Gender differences between patients experiencing problems while in hospital.

Question	Male (*n *= 61), *N* (%)	Female (*n *= 21), *N* (%)	95% *p *< 0.005 *X* ^2^
What problems do you experience			
Restrictions and freedom	33 (54)	10 (48)	*p *= 0.608
Boredom	35 (57)	8 (38)	*p *= 0.127
Health	24 (39)	12 (57)	*p *= 0.156
Bullying	14 (23)	7 (33)	*p*= 0.347
Being discharged	17 (28)	8 (38)	*p *= 0.379
Family	19 (31)	9 (43)	*p *= 0.329
Confidentiality/dignity	14 (23)	5 (24)	*p *= 0.935
Employment	8 (13)	1 (4)	*
Gender identity	7 (11)	2 (8)	*
Debt/money	5 (8)	3 (12)	*
Drug/addiction	4 (6)	0 (0)	*
Who do you prefer to talk to when you have a problem?			
Staff	47 (77)	16 (76)	*p *= 0.935
Family	37 (61)	13 (62)	*p *= 0.919
Other patients	25 (41)	5 (24)	*p *= 1.587
Friends	20 (33)	4 (19)	*p *= 0.232
Chaplaincy	8 (13)	7 (33)	*p *= 0.0387
Problems are			
Ongoing	27 (44)	12 (57)	*p *= 0.308

* count too small for calculation

### 3.2 Review of patient and staff feedback on the workbook materials

Twenty‐two patients and 49 staff from across the whole hospital site reviewed the materials. For staff, this included mainly females (*n* = 40, 81%), representing a range of professional groups: qualified nurses (*n* = 13, 26%); health care assistants, student nurses and nursing associates (*n* = 41, 83%); psychology/psychotherapy staff (*n* = 4, 4%); occupational therapists (*n* = 2, 4%) and consultant psychiatrists (*n* = 2, 4%). Representing staff working on female‐only wards (*n* = 13, 26%); male‐only wards (*n* = 27, 55.1%) and no specified ward (*n* = 9, 18%). The majority of those who completed the feedback provided some qualitative comments (*n* = 39, 71%).

Comments on the acceptability of the workbook were generally positive from hospital staff and patients,

‘Overall it is a good workbook; could make an easy read version for those who can't understand this booklet’; ‘I think the length of time was good’; ‘I think it will help other people’; ‘Easy to understand’; “insightful’.

‘Is bright and colourful’; ‘I think it's really good for patients, it has nice colours and easy to understand’; ‘makes assumptions on emotional literacy’;

Some staff felt that the wording in the workbooks could be further refined; particularly concerning the use of language and the number of words used:

‘the wording could be simplified for some individuals working on admission ward with acutely unwell patients may struggle to process the amount of wording use’.

The portrayal of the case studies characters devised with the patients was positively received by staff, ‘I personally enjoyed the case study and is typical of what some service users experience’; although the imagery used to depict the characters warranted further improvement ‘the images used to represent the characters could be further improved for this patient group’.

How patients would complete the workbook (i.e., with staff; or alone) was not specified in the feedback brief. Staff reflected upon whether patients would need support from colleagues to complete the workbook or whether this was something they could do alone; mixed responses were received,

‘depending on the individual it would determine whether they would need staff to support them’;

There were also some concerns that the model maybe perceived by patients negatively; this suggested that further work on the presentation of step one was required: ‘Step one needs to be delivered with care as telling someone they have a negative attitude could come across as blaming or dismissive’.

The quantitative feedback from the staff (Table 2) highlighted further considerations in relation to the visual presentation of the characters (acceptability); use of language (accessible) and readability (accessibility, acceptability, and feasibility) and use of the workbook in solving problems in the future. No significant differences at *p* < 0.05 were identified between hospital staff and patients (Table 2).

**Table 2:** Overall patient and staff responses to the review of the workbook.

Patient responses (*n* = 22)		Staff responses (*n* = 49)			
Item on the survey	Overall score (min 5 to max 110)	Mean score and range	Overall score (min 5 to max 110)	Mean score and range	Statistical differences (*p *< 0.05)
The character in the workbook is relatable	86	4.09 (3–5)	201	4.10 (1–5)	2.62, *p* = 0.268
The image of the character is appropriate	86	4.09 (2–5)	196	4.0 (1–5)	0.577, *p* = 0.749
The language in the workbook is easy to understand	87	4.14 (3–5)	200	4.08 (2–5)	2.22, *p* = 0.329
The amount of text in the workbook is appropriate	80	3.80 (2–5)	195	3.97 (2–5)	0.679, *p* = 0.711
The workbook would be helpful in helping me solve problems in the future	85	4.04 (3–5)	212	4.32 (2–5)	2.156, *p* = 0.340

Abbreviations: max, maximum; min, minimum.

## 4 DISCUSSION

This study represents the adaptation of a survey and the co‐production of two case studies for use with a problem‐solving workbook. The process involved engagement from patients, hospital staff and the research team who used the MRC framework as a transparent methodical approach.

The findings emphasised the premise that adapted interventions are more likely to be more acceptable and feasible when co‐production has taken place.^22^ Use of the novel character case study methodology enabled engagement with a group of patients where adherence is often an issue^21^ and where they might otherwise struggle to access research engagement.^32^ Such opportunities are particularly important for patients who are naturally vulnerable and already socially excluded from the rest of the population. The process identified the important use of appropriate language to help facilitate a valued engagement process where individuals feel that their preferences are respected and prioritised was an important part of this process.^35^ Patients engaging with the survey adaptations and the co‐production of the case studies used their own words to help contextualise their lived experiences and the journey within the hospital walls, adding to credibility and acceptability of the workbook.

### 4.1 Research implications

Co‐production is a necessary and important consideration of intervention design and development. Further refinement of the workbook is required to improve the visual representation of the case study characters; to understand the mechanisms for implementation (patients were more likely to share problems with staff than their peers), the timing of intervention delivery (patients nearing discharge were more likely to use the skills to help to support with the transition process) and use of appropriate language (requiring feedback from speech and language therapists). A high‐quality feasibility randomised controlled trial to measure recruitment rates, intervention adherence and attrition would be the next proposed step along with measures to identify whether such an intervention may lead to reduced length of stay in such services.

### 4.2 Study limitations

The study was not without its limitations, firstly, it was a small‐scale study limited by the volunteer, self‐selecting nature of those who chose to take part. The small nature of this sample makes it unlikely to be representative of those who spend time in secure mental health hospitals. Furthermore, we did not collect any data on those who did not want to take part, and a proportion of those were deemed too unwell to take part. Learning showed that extensive staff support was required to motivate and encourage people from different ethnic minority backgrounds to take part. For this reason, some staff supported the attendance of patients at the workshops where the command of the English language was not sufficient. Efforts were made to minimise any bias by sense‐checking the verbal comments made by patients and staff. Researchers read the survey and feedback sheets to patients to ensure parity of access to the materials; this may have led to more socially desirable responses from the patients themselves. The disproportionate number of staff in comparison to the numbers of patients who completed the feedback; was primarily related to the project resources and the time taken to engage with this patient group.

PSS do form a necessary part of everyday life; use and maintenance of these skills are vital in providing life skills that aid proactive decision‐making leading to positive actions, especially at times of transition.^16^, ^18^ Such skills promote the opportunity to develop and improve appropriate coping strategies that empowers patients to have an element of 'control' over their own circumstances. Patients in the transition process to discharge (and having spent a long time in the hospital) are likely to face complex challenges with housing, employment, relationships and budgeting. Development and maintenance of this skill set is important to enable patients to contribute to their life own choices both while in hospital and in the lead up to discharge from the hospital to produce a successful transition process.

## 5 CONCLUSIONS

Co‐production is a worthwhile and important element of intervention design. The adapted problem‐solving workbook was acceptable to patients and staff. Further development of the intervention is required to support a large‐scale evaluation of the use of PSS with this population.

## AUTHOR CONTRIBUTIONS

**Amanda E. Perry**: Conceptualisation; investigation; funding acquisition; writing ‐ original draft; methodology; validation; writing—review and editing; project administration; formal analysis; resources; supervision; data curation. **Heather Baker**: Conceptualisation; investigation; writing—original draft; methodology; writing—review and editing; project administration. **Anne Aboaja**: Conceptualisation; investigation; funding acquisition; writing— review and editing. **Lindsey Wilson**: Writing—review and editing; project administration; methodology. **Sarah Morris**: Writing—review and editing. **Patient Public Involved Service User**: Review and editing.

## CONFLICT OF INTEREST STATEMENT

The authors declare no conflict of interest.

## ETHICS STATEMENT

We sought advice from the local Trust Research and Development Office. The consultation according to the [https://www.hra-decisiontools.org.uk/ethics/] guidance states that no ethical approval was required as part of this process. Nevertheless, all data collection was accompanied by a statement of informed consent, and all responses were anonymised. By taking part in the study participants agreed to give consent for their anonymous data to be used and stored by members of the research team. The responses will be destroyed after 5 years and in accordance with General Data Protection Regulations.

## Supporting information

Supporting information.

## Data Availability

Anonymised data is available by author request.
