# Supplementary material for: Co‐production and adaptation of a prison‐based problem‐solving workbook to support the mental health of patients housed within a medium‐ and low‐secure forensic service
Source: Health Expect. 2024 Feb 23;27(2):e13997. doi: 10.1111/hex.13997 (PMC10891435; doi:10.1111/hex.13997)
Supplement: Supplementary file 1 — Supporting information. [file HEX-27-e13997-s001.docx]

Supplementary materials

**Appendix A: Original prison survey**

**PROBLEM SOLVING CHAMPIONS TEAM**

**PLEASE COMPLETE THE FOLLOWING INFORMATION. THIS IS YOUR CHANCE TO HAVE YOUR SAY.**

**THIS WILL ONLY TAKE A FEW MINUTES TO COMPLETE. PLEASE HAND IT BACK TO THE PROBLEM-SOLVING CHAMPION ON YOUR WING**

1. Have you heard about the problem-solving scheme? YES NO
2. Do you know who your champion is on your wing? YES NO
3. Have you spoken to a problem-solving champion? YES NO
4. Have you experienced any problems whilst in prison? YES NO
5. What type of problems do you worry about in prison?

- Housing
- Employment
- Bullying
- Family Relationships
- Physical/Mental Health
- Debt/Money
- Drugs/Addiction

*(Please tick those which apply to you)*

1. Who do you talk to in the prison about your problems

- HMPPS Staff
- A Listener
- A Problem Solving Champion
- Another prisoner
- Chaplaincy
- Probation/Offender Supervisor

*(Please tick those which apply to you)*

1. How often does your problem(s) occur?

- Ongoing/Unresolved problem
- Frequent problem (twice a month)
- Rarely (once every 3-6 months)

*(Please tick those which apply to you)*

**Appendix B: New adapted survey to identify problems experienced by patients in secure hospital settings.**

The data collected in this survey will be used to help us develop training materials that will help people cope better with the problems they might experience in hospital. By completing this survey, **you are giving consent for your anonymous data** to be used and stored by members of the research team. The responses will be destroyed after a 5-year period and in accordance with General Data Protection Regulations.

| **Gender** *(please tick)* | Male  (including trans male)  ☐ | | Female  (including trans female)  ☐ | | Other ☐ | | Prefer not to say ☐ |
| --- | --- | --- | --- | --- | --- | --- | --- |
| **Age range** *(please tick)* | 18-24  ☐ | 25-34  ☐ | 35-44 ☐ | 45-54 ☐ | 55-64 ☐ | 65+      ☐ | Prefer not to say ☐ |
| **Ethnicity** *(please tick)* | White ☐ | Mixed ☐ | Asian/Asian British ☐ | Black/Black British ☐ | Arab    ☐ | Other ☐ | Prefer not to say    ☐ |
| **In which ward are you currently based?** |  |  |  |  |  |  | |
| **How long have you been in hospital?** |  |  |  |  |  |  | |

**Have you experienced any problems whilst in hospital?** *(Please tick)*

Yes ☐   No☐

1. **What type of problems do you worry about in hospital?** *(Please tick all which apply to you)*

Housing ☐    Employment ☐   Bullying ☐   Family relationships ☐   Physical/mental health ☐

Debt/money ☐   Drugs/addiction ☐   Boredom ☐   Being discharged ☐  Gender identity  ☐

Restrictions/Freedom  ☐   Confidentiality/dignity ☐   Other *(please state)* ☐*…………………………………………………………………………………………………………………………………….*

1. **Who do you talk to in hospital about your problems?** *(Please tick all which apply to you)*

Staff ☐   Family ☐   Friends ☐   Another patient ☐   Chaplaincy ☐   Other *(please state)* ☐*…………………………………………………………………………………………………………………………………….*

1. **How often does your problem(s) occur?** *(Please tick)*

Ongoing/unresolved ☐   Frequent problem (twice a month) ☐   Rarely (every 3-6 months) ☐

**Appendix C: Original Male character in custody**

James is 22 years old. He is one of six siblings and lives with his father and stepmother. James’s relationship with his parents was troubled from a young age and his father would come home drunk and beat James. His stepmother found it difficult to deal with James’s aggressive emotional outbursts and he was excluded from school at age 11 for poor behaviour and angry outbursts. James started to mix with a gang of older boys who were known in the area for committing petty crimes. He became involved in drugs at age 13 years and was caught by the police for burglary when he was 16 years old. He also had a series of relationships with older women which led to a number of pregnancies resulting in two sons and a new baby. James’s stepmother was unable to control his behaviour and did not want him in the house anymore so James was asked to leave.

James went to stay with a friend but soon ended up living in a hostel. He found it difficult to get a job and ended up stealing to support his drug habit. His physical and mental health deteriorated and he no longer took care of himself. One day he took drugs and alcohol and ended up in the Accident and Emergency department at the hospital. James was finally convicted for a series of burglaries and ended up in prison for the first time. At an all-time low James has contact with his family, with his partner visiting him with the kids on a regular basis. James regularly self-harmed when he was feeling particularly stressed in prison. James was placed in a shared cell and though having settled into prison life James felt angry and frustrated. On a recent prison visit his partner told him that the council were planning to change their accommodation because James was no longer living with them. The change in circumstances would mean that James’s partner could be moved outside of the local area. James returned to the wing in a low mood. He feels inadequate and powerless to do anything about the change in circumstances. James ‘kicks off’ in his cell.

**Original female character in custody**

Janice is a 48 year old woman who lives with her present husband who has not worked for several years. Janice looks after her teenage son. She has an older daughter who is a single parent with a little boy. Janice has had a difficult life; her parents threw her out of her home when she was 15 and pregnant with her first child. The baby girl was adopted but Janice struggled to cope afterwards. She began binge drinking on a weekend, had one-night stands and a series of casual relationships. At her worst times Janice took several overdoses of paracetamol which required hospital treatment. Aged 23, Janice later was in a relationship with a violent partner with whom she had a second daughter but left her partner after 7 years She continued to raise her daughter and later married and settled with a new partner, they had a son and she found work as a community carer.

With a growing son, Janice’s relationship with her daughter became very stormy resulting in the daughter moving out of the family home. This brought back all the memories of Janice’s own early life. To cope, Janice started drinking and had a phase of taking overdoses. The problems became more difficult to deal with when Janice’s husband lost his job. He began to drink heavily and became depressed. Janice took on extra work overtime Janice found it increasingly difficult to cope as the cost of fuel bills rose, and she had rising debts. Janice started stealing for the people she cared for. Over three years Janice had managed to remove £6,500. Janice was finally caught and received a sentence of 18 months imprisonment. Janice felt intimidated by the surroundings and the other prisoners. In a distraught state and without alcohol to calm her, Janice cut herself with broken teacup.

**Appendix D: Patient and staff consultation form on the workbooks**

By completing this survey, **you are giving consent for your anonymous data** to be used and stored by members of the research team. The responses will be destroyed after a 5-year period and in accordance with General Data Protection Regulations. Please help us to evaluate the problem-solving workbook you have seen today by indicating the degree to which you agree with each statement.

**Please circle your response**

|  | Strongly Disagree | Disagree | Neither Agree nor Disagree | Agree | Strongly Agree |
| --- | --- | --- | --- | --- | --- |
| 1. The character in the workbook is relatable | 1 | 2 | 3 | 4 | 5 |
| 2. The image of the character is appropriate | 1 | 2 | 3 | 4 | 5 |
| 3. The language in the workbook is easy to understand | 1 | 2 | 3 | 4 | 5 |
| 4. The amount of text in the workbook is appropriate | 1 | 2 | 3 | 4 | 5 |
| 5. The workbook would be useful in helping me solve problems in the future | 1 | 2 | 3 | 4 | 5 |

1. Any other comments:
